# Modification of human metallothioneins by garlic organosulfur compounds, allicin and ajoene: direct effect on zinc homeostasis with relevance to immune regulation

**DOI:** 10.1007/s10534-025-00716-3

**Published:** 2025-07-30

**Authors:** Karolina Mosna, Alicja Orzeł, Michał Tracz, Sylwia Wu, Artur Krężel

**Affiliations:** 1 Department of Chemical Biology, Faculty of Biotechnology, University of Wroclaw, Joliot-Curie 14a, 50-383 Wrocław, Poland; 2 Laboratory of Protein Mass Spectrometry, Faculty of Biotechnology, University of Wroclaw, Joliot-Curie 14a, 50-383 Wrocław, Poland

**Keywords:** S-thioallylation, Labile zinc, Protein modification, Zinc homeostasis, Reactive species

## Abstract

Garlic (*Allium sativum*) is a key culinary ingredient and agricultural product that releases its pungent aroma when cut or crushed due to the presence of volatile organosulfur compounds. These compounds, specifically allicin and ajoene, rapidly modify human metallothioneins (MTs), leading to the dissociation of Zn(II) ions. This study employed a top-down protein MS analysis to identify the most reactive cysteine sites in MT2, confirming that both compounds induce S-thioallylation and alter Zn(II) binding. Spectrophotometric studies indicated that both compounds mobilized nearly six molar equivalents of Zn(II) from MT2 after one hour, with ajoene demonstrating slightly higher dissociation efficiency than allicin. Moreover, allicin mobilized Zn(II) from all human isoforms of MT (MT1-MT4), with the lowest efficiency observed for MT4. Mass spectrometry analysis revealed a diverse array of modified complexes, with ajoene resulting in more complex formations than allicin. Reduced glutathione interacts with both untreated and allicin- or ajoene-treated MTs, forming mixed and partially oxidized complexes, highlighting the relationship between these compounds depending on the cell’s redox state. The use of reducing agents demonstrated that MTs’ S-thioallylation is reversible, allowing the protein to rebind Zn(II) after reduction, thereby maintaining its role in zinc homeostasis and rendering the effects of allicin temporary. The health implications of the modifications are significant, given the established role of Zn(II) in inhibiting pro-inflammatory pathways and enhancing antioxidant responses. Zn(II) ions were shown to inhibit the activation of the NF-κB pathway, which regulates pro-inflammatory cytokines. Our findings indicate that the modification of metallothioneins (MTs) by garlic-derived organosulfur compounds may play a crucial role in zinc homeostasis, with implications for immune regulation in humans, and warrant further investigation into their potential therapeutic applications.

**Graphical Abstract:**

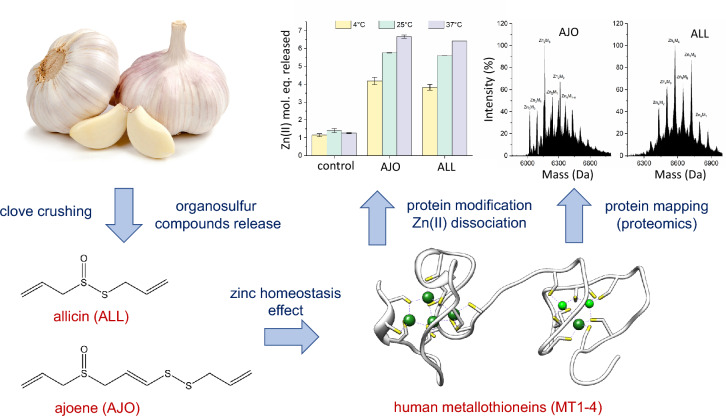

**Supplementary Information:**

The online version contains supplementary material available at 10.1007/s10534-025-00716-3.

## Introduction

Garlic (*Allium sativum*) is globally recognized both as a culinary staple and a major agricultural product. Cutting or crushing garlic cloves releases its characteristic pungent aroma caused by volatile sulfur compounds produced when the tissue is disrupted (Borlinghaus et al. 2021). These compounds defend garlic against pathogens and pests and exhibit antibacterial properties (Cavallito and Bailey 1944). The primary odor compound is allicin (diallyl thiosulfinate, ALL) (Cavallito et al. 1944), produced from odorless alliin (S-allylcysteine sulfoxide) by alliinase, released during tissue damage (Lawson et al. 1991; Lancaster and Collin 1981). Allicin constitutes 60–80% of the garlic organosulfur compounds (GOSC) formed, with a single garlic clove producing up to 5 mg (Lawson and Hughes 1992). The absorption and metabolism of allicin and its derivatives are not fully understood, but some decomposition likely occurs in the stomach, forming volatile compounds like diallyl sulfide (DAS) and diallyl disulfide (DADS), which further metabolize into compounds such as allyl methyl sulfide (AMS) and allyl mercaptan. These have been detected in human breath after garlic consumption (Minami et al. 1989; Rosen et al. 2001; Suarez et al. 1999; Trio et al. 2014). However, allicin and its derivatives, like ajoene (AJO) and vinyldithiins, have not been found in human blood, urine, or stool (Lawson 1998). In contrast, S-allyl cysteine (SAC) forms during garlic extract maturation in aqueous ethanol (Elosta et al. 2017).

**Fig. 1:**
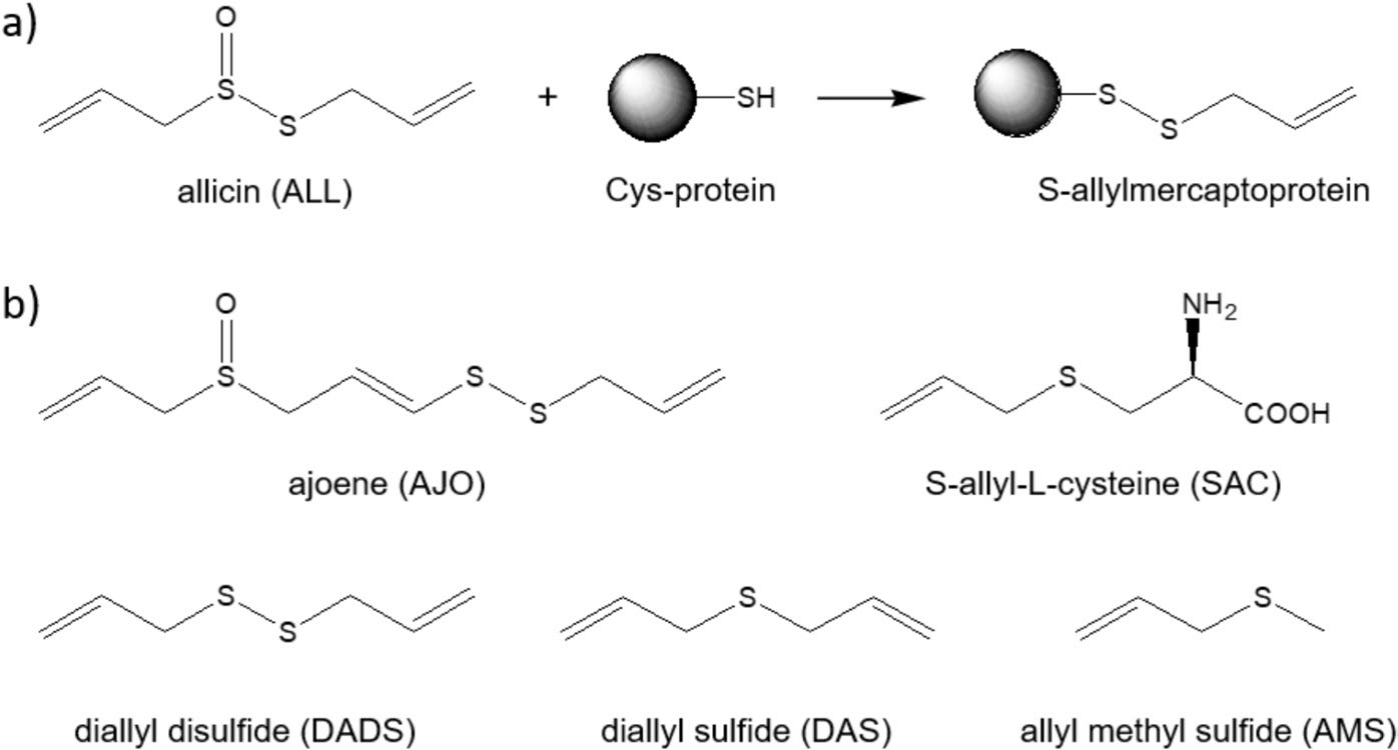
Garlic organosulfur compounds (GOSC). **a** Reaction of allicin with Cys-containing protein. **b** Structures of other GOSC used in this study with their abbreviations

Allicin, a reactive GOSC, quickly reacts with reduced glutathione (GSH) in an S-thioallylation reaction, forming S-allylmercaptoglutathione (GSSA) (Rabinkov et al. 1998, 2000; Schafer and Buettner 2001). It also reacts with accessible protein thiols (Fig. 1a), which is central to its biological activity. Identifying proteins that interact with allicin and its metabolites is important due to garlic consumption, inhaling its vapors, and its growing use in plant protection, especially given its reported health benefits (Slusarenko et al. 2008). Mass spectrometry-based proteomics has shown that allicin modifies proteins like tubulin (TUBB, TUBA1), actin (ACTG), cofilin (CLF1), superoxide dismutase-1 (SOD-1), elongation factors (EEF2), heat shock proteins (HSP90, HSPA4), and glycolysis enzymes (ALDOA, GAPDH, PKM) in Jurkat cells. It also causes Zn(II) dissociation from zinc proteins like SOD-1 in EL-4 cells (Gruhlke et al. 2019). Allicin and ajoene affect cell cycle arrest and apoptosis in cancer cells. Allicin blocks the cell cycle at the S/G2/M phase and induces apoptosis in leukemic cells (Trio et al. 2014), while ajoene halts the cycle at G2/M and induces apoptosis in HL-60 cells (Powolny and Singh 2008). Ajoene also upregulates NAD(P)H:quinone oxidoreductase-1 (NQO1) by activating ERK and Nrf2 in non-tumorigenic MCF-10A cells, enhancing antioxidant responses (Cho et al. 2019). In CT26 tumor-bearing mice, ajoene inhibits muscle degradation by downregulating Jak/STAT3 and SMADs/FoxO (Lee et al. 2019).

Metallothioneins (MTs) are small, cysteine-rich proteins primarily involved in the metabolism of Zn(II) and Cu(I) ions (Krężel and Maret 2017, 2021; Blindauer and Leszczyszyn 2010; Babula et al. 2012). They also detoxify harmful Cd(II), Pb(II), and Hg(II) ions (Scheller et al. 2018; Freisinger and Vašák 2013). Mammalian MTs consist of four main isoforms (MT1-MT4, 6–7 kDa) and several MT1 subisoforms, found in various cell compartments and differing in metal-binding properties and tissue localization (Krężel and Maret 2021; Apostolova et al. 1999; Pountney et al. 1994; Quaife et al. 1994; Uchida et al. 1991; Ye et al. 2001). MTs are 60 to 68 amino acids long, forming a dumbbell-shaped polypeptide with two thiol-rich regions, the α- and β-domains, linked by a conserved -Lys-Lys-Ser- linker in isoforms MT1-3 and -Arg-Lys-Ser- in isoform MT4 (Krężel and Maret 2021; Singh et al. 2023; Braun et al. 1992; Yuan et al. 2023). MTs bind up to seven Zn(II) ions with varying affinities, from nano- to picomolar, playing a key role in zinc buffering and maintaining Zn(II) levels in mammalian cells (Kocyła et al. 2021; Pomorski et al. 2023; Krężel and Maret 2021; Artells et al. 2013; Drozd et al. 2018; Peris-Díaz et al. 2020). Due to their high cysteine content, MTs are highly reactive, depending on factors like isoform, bound metal ions, and interacting molecules (Krężel and Maret 2021). They interact with oxidized glutathione (GSSG) and synthetic disulfides such as DTNB (5,5′-dithiobis(2-nitrobenzoic acid, Ellman’s reagent), forming mixed disulfides or oligomers (Savas et al. 1993; Chen and Maret 2001; Haase and Maret 2008). Oxidation of Zn(II)/S clusters or reaction with reactive oxygen/nitrogen species leads to partial Zn(II) dissociation due to structural changes and lack of Zn(II) affinity to disulfides (Krężel and Maret 2007a). Selenium compounds, like ebselen, cause full oxidative modification and Zn(II) dissociation (Chen and Maret 2001). Alkylating agents, such as melphalan and chlorambucil, also modify MTs, resulting in metal dissociation (Yu et al. 1995; Antoine et al. 1998). MT oxidation may be reversible, allowing for Zn(II) rebinding, though alkylation is typically irreversible (Maret and Vallee 1998). Zinc signaling is critical for homeostasis, regulated by zinc transporters that increase (Zip family) and lower cytosolic zinc (ZnT family) (Maywald et al. 2017). During infection, inflammation causes Zn(II) to shift from serum to cells, especially in the liver, signaling the immune system to respond. This Zn(II) influx increases MT expression, neutralizes ROS/RNS, and activates zinc finger transcription factors responsible for IL-2 production and T cell activation (Maywald et al. 2017; Prasad 2008; Korkola and Stillman 2023; Aravindakumar et al. 1999; Thornalley and Vasák 1985). Zinc deficiency can lead to immune dysfunction, diabetic nephropathy, and impaired wound healing (Nie et al. 2022; Prasad 2008; Wessels et al. 2021). Therefore, ensuring sufficient zinc intake through foods like red meat, fish, seafood, oats, or nuts is crucial (Maret and Sandstead 2006).

Previous proteomic studies have shown that allicin modifies many zinc proteins, but metallothioneins were not detected in Jurkat cells (Gruhlke et al. 2019). This may be due to their low molecular mass (the smallest identified protein was 10 kDa) or multicysteine residue modifications, which complicate tryptic peptide analysis. The same study reported modifications in several transcription factors with zinc finger motifs, which are less reactive than metallothioneins (Kluska et al. 2018). This raised interest in whether human metallothioneins, which are highly reactive and Cys-rich, undergo S-thioallylation by GOSC (Fig. 1b), and whether this leads to Zn(II) dissociation. The results presented here offer new insights into the function of metallothionein and the roles of zinc and garlic in human health.

## Materials and methods

### Chemicals and reagents

E/Z-Ajoene, diallylsulfide (DAS), S-allylcysteine (SAC or L-deoxyalliin) were from Cayman Chemical. Hydrogen peroxide (30%), allyl methyl sulfide (AMS), ethylenedinitrilotetraacetic acid (EDTA), Trypsin MS grade, 2-carboxy-2′-hydroxy-5′-sulfoformazylbenzene monosodium salt (Zincon), diallyl disulfide (DADS), NaClO_4_, anhydrous MgSO_4_ were purchased from Sigma-Aldrich. The metal-chelating resin Chelex 100® was from BioRad. HCl, Tris base, dimethyl sulfoxide (DMSO), Water MS grade, Formic acid (98%) LC–MS grade, Ammonium acetate, n-hexane, and dichloromethane (DCM) were from VWR Chemicals. For top-down LC–MS all solvents were from Witko (Łódź, Poland), and GluFib B was from Waters (Milford, MA). HCl (trace metal grade), 4-(2-pyridylazo)resorcinol (PAR), NaOH, ammonium solution 25%, were obtained from Merck Millipore. 5,5′-Dithiobis(2-nitrobenzoic acid) (DTNB) was from Tokyo Chemical Industry. Methanol LC–MS grade and ethyl acetate were from Witko (Łódź, Poland). Tris(2-carboxyethyl)phosphine hydrochloride (TCEP) was purchased from Iris Biotech GmbH (Marktredwitz, Germany). CD3OD (D, 99,8%) was from Cambridge Isotope Laboratories Inc. Tryptone, yeast extract, Terrific Broth (TB), agar, isopropyl-β-d-1-thiogalactopyranoside (IPTG) was purchased from Lab Empire. Glycerol was from STANLAB (Lublin, Poland). Ampicillin, chloramphenicol, 1,4-dithiothreitol (DTT), 4-(2-hydroxyethyl)piperazine-1-ethanesulfonic acid sodium salt (HEPES) were from Roth, pTYB21 vector, Gibson Assembly® Cloning Kit, restriction enzymes and chitin resin from New England BioLabs. Aqueous solutions were configured with Milli-Q water (18.2 MΩ·cm^−1^, 0.22 μm filter). Microporous membrane filters (0.22 and 0.45 μm) were used for further purification (Jet Biofil, China). All reagents were purchased from commercial suppliers and used accordingly. All buffers were prepared with Milli-Q water obtained with a deionizing water system (Merck). To eliminate trace metal ion contamination, all pH buffers were treated with Chelex 100® resin and degassed over 2 h prior use. The concentration of stock solutions of metal ion salts was 0.05 M, confirmed by a representative series of ICP-MS measurements. For the culture of *E. coli*, Luria–Bertani (LB) medium and agar plates were used.

### Preparation of allicin

Allicin synthesis was based on the protocol by Albrecht et al. (2017) with some modifications. In the initial step, formic acid (>98%) was mixed with hydrogen peroxide (30%) in a 3:5 (v/v) ratio and incubated at room temperature for 90 min. Diallyl disulfide (DADS) (98%) was dissolved in methanol (99%) in a ratio of 1:5 (v/v) and stirred for 5 min on ice. Then, the peracid mixture was added slowly, drop by drop, to DADS to achieve a final ratio of 2:3 (v/v), and stirring on ice was continued for 15 min. A tenfold excess of Milli-Q water was added to stop the reaction. The mixture was extracted three times using dichloromethane (DCM). Anhydrous MgSO_4_ was added to the extracted liquid to remove the remaining water. After filtration, the solvent was evaporated under reduced pressure and redissolved in a mixture of n-hexane and ethyl acetate (2:1, v/v). Purification was performed by FLASH chromatography using a PLC 2020 Personal Purification System (Gilson) with a PuriFlash column (130 × 21 mm, SIHP-JP, 30 μm) and a solvent mixture of n-hexane and ethyl acetate (2:1, v/v). Fractions were collected into tubes cooled in an ice bath, and the solvents were removed under reduced pressure at room temperature. The resulting oil substance was stored at −80 °C. The identity of allicin was confirmed using NMR (Bruker Avance III 500 MHz). For this purpose, the allicin sample was dissolved in methanol-D4, and the 1H spectrum was recorded at 298 K using a Bruker Avance III 500 spectrometer. The concentration of allicin was measured using a molar extinction coefficient of ε_240_ = 2,380 (M^−1^·cm^−1^) (Lawson and Wang 2001).

### Production and purification of human MTs

Metallothioneins were produced, as shown in the report by Mosna et al. (2023). BL21(DE3)pLysS *E. coli* cells were transformed using expression vectors with an intein protein tag and cultured in a TB culture medium at 37 °C until reaching 0.6 OD_600_. Induction was performed with 0.1 mM IPTG and 0.3 mM ZnCl_2_ overnight at 20 °C with vigorous shaking. Subsequently, cells were centrifuged at 4 °C, 4,000*g* for 15 min, then resuspended in cold buffer A (20 mM HEPES-Na^+^, pH 8.0, 500 mM NaCl, 1 mM TCEP) and sonicated three times for 15 min each. The supernatant was separated from the pellet by centrifugation at 4 °C, 16,000*g*, and incubated with chitin resin overnight with mild shaking at 4 °C. The resin was washed with buffer A until the absorbance at 280 nm of the flow-through was below 0.1. For the cleaving step, 40 mL of 20 mM HEPES-Na^+^, pH 8.0, 500 mM NaCl, and 100 mM DTT buffer were used, and the process was carried out for 48 h at room temperature. The protein was concentrated using Amicon Ultra-4 Centrifugal Filter Units with a 3 kDa membrane cutoff (Merck) and then acidified to a pH of 2.2. Metal-free metallothionein was purified on either a HiLoad 16/600 or a Superdex 75 increase 10/300 column (GE) in 10 mM HCl. Mass confirmation was done using ESI–MS qTOF Bruker Maxis Impact mass spectrometer. MTs reconstitution was carried out using ZnSO_4_ under a nitrogen blanket, and the pH was adjusted to 8.6 with a 1 M solution of Tris base. Concentrated proteins were purified on a HiLoad® 16/600 Superdex® 75 pg column (GE) in 20 mM Tris–HCl pH 8.6. The concentrations of Zn(II) and thiolates were determined using PAR and DTNB assays, respectively (Kocyła et al. 2015; Eyer et al. 2003). The proteins were stored at −80 °C.

### Spectrophotometric study of Zn(II) dissociation with PAR

All experiments with PAR were conducted using a JASCO V-650 spectrophotometer with a Peltier module ETCS-761 and a buffer of 50 mM HEPES-Na^+^, 100 mM NaCl, pH 6.5, 7.4, or 8.5 in a 1 cm quartz cuvette. To study Zn(II) dissociation upon addition of garlic compounds, a 100 µM PAR solution was mixed with 40–160 µM allicin (49 mM stock in water), ajoene (100 mg/mL stock in ethyl acetate provided by the vendor), or 80 µM AMS (10 mg/mL stock in water), DAD (5 mg/mL stock in DMSO), DADS (5 mg/mL stock in DMSO), or SAC (10 mg/mL stock in water). Then, a particular MT isoform was added to achieve a final concentration of 1 µM, and absorbance at 492 nm was measured in a time-dependent mode for 60 min at 4, 25, or 37 °C. In the case of Zn(II) dissociation study without PAR initially, MT2 samples were first incubated with 80 µM ajoene and allicin for a certain period of time, ranging from 5 to 120 min, before adding PAR to achieve a final concentration of 100 µM. Molar Zn(II) equivalents transferred to PAR were calculated based on recorded absorbances and the molar extinction coefficient of the ZnH_x_(PAR)_2_ complex for pH 6.5, 7.4, and 8.5, which are respectively 49,900, 71,500, and 86,100 M^−1^·cm^−1^ (Kocyła et al. 2015).

### Spectrophotometric study of S-thioallylation reversibility with Zincon

Zincon (2-carboxy-2′-hydroxy-5′-sulfoformazylbenzene monosodium salt) instead of PAR was chosen due to its significantly lower to Zn(II) affinity (Kocyła et al. 2017). All experiments with Zincon were conducted using a JASCO V-650 spectrophotometer equipped with a Peltier module (ETCS-761) and a buffer of 50 mM HEPES-Na^+^, 100 mM NaCl, pH 7.4, in a 1 cm quartz cuvette. First, the increase in absorbance at 618 nm, indicating the transfer of Zn(II) from 1 µM MT2 to 100 µM Zincon under the influence of 80 µM allicin, was monitored for one hour. After this period, 1 mM of the reducing agent TCEP was added, and the measurement continued for 30 min. Control measurements involved continuous monitoring of absorbance after sequential addition to the cuvette of 100 µM Zincon, 3 µM ZnSO_4_, 80 µM allicin, and 1 mM of the reducing agents GSH, DTT, and TCEP. Molar Zn(II) equivalents transferred to Zincon were calculated based on recorded absorbances and the molar extinction coefficient of the ZnH_x_(Zincon) complex, which is 24, 200 M^−1^⋅cm^−1^ (Kocyła et al. 2017).

### Circular dichroism (CD)

CD spectra of Zn(II)-loaded MT2 were recorded using a J-1500 Jasco spectropolarimeter (JASCO) at 25 °C in a 2 mm quartz cuvette under a constant nitrogen flow over the range of 200–300 nm with a 200 nm⋅min^−1^ speed scan. Final spectra were averaged from three independent scans, measured every 10 min until 1 h of reaction time was reached, and analyzed using Jasco Spectra Manager software. The results are presented as molar ellipticity [Θ] in deg⋅cm^2^⋅dmol^−1^ units. The concentration of MT2 used for CD measurements was 10 µM in 20 mM Tris–HCl, 100 mM NaClO_4_, pH 7.4 with the addition of 800 µM allicin or 5 µM in 20 mM Tris–HCl, 100 mM NaClO_4_, pH 7.4 with the addition of 400 µM ajoene. The differences in the concentrations used in the samples with allicin and ajoene were due to the generation of higher voltage on the detector after the addition of ajoene, which limited the maximum applicable concentration of ajoene to 400 µM.

### Mass spectrometry

ESI–MS experiments were performed on a quadrupole time-of-flight (qTOF) Bruker Maxis Impact mass spectrometer (Bruker Daltonik GmbH) calibrated with a commercial ESI-TOF tuning mix (Sigma-Aldrich). Samples were directly infused with a 3 μl/min flow rate. ESI–MS spectra were recorded in positive mode with a capillary voltage of 2 kV, end plate offset potential of 500 V, nebulizer gas (N_2_) pressure of 1.5 bar, drying gas (N_2_) flow rate of 4 l/min, and drying temperature of 180 °C. The mass range was set from 100 to 3000 m/z and recorded and averaged over 1 min. The Bruker Compass data analysis software package was used for data processing. Samples with apo-MTs were measured in solution water/methanol/formic acid (50/50/0.1). Complexes of holo-MTs with Zn(II) were prepared in 50 mM ammonium acetate, pH 7.4. The samples, after reaction with modificators were purified two times using Zeba™ Spin Desalting Columns 7 K MWCO (ThermoFischer Scientific). For measurements with DTT, GSH, and TCEP, a fivefold excess of these reagents was used relative to the cysteine residues. To remove the excess reducing agents, a threefold buffer exchange was performed using 3 kDa Amicon filters.

### Identification of S-thioallylated Cys residues in Zn_7_MT2 by top-down mass spectrometry

In order to identify the most reactive site in MT2, a lower concentration of ajoene and allicin was used so that the concentration ratio of these compounds to the concentration of cysteine residues was 1:1. This allowed for the modification of one site per MT2 molecule. The reaction was carried out in 50 mM ammonium acetate, pH 7.4, for 15 min. After this time, the samples were analyzed with an M-Class Acquity UPLC system coupled to a Synapt XS HRMS equipped with an ESI ion source interface (Waters). Mobile phase A consisted of H_2_O + 0.1% formic acid (FA), 0.05% trifluoroacetic acid (TFA), while mobile phase B consisted of acetonitrile + 0.1% FA, 0.05% TFA. 2 pmol of MT2 in 50 mM ammonium acetate were injected, desalted on-system, and a five minute 15–80% B linear gradient was applied for sample separation on a nanoEase M/Z BEH C4 300 Å, 5 μm, 300 μm × 50 mm column (Waters), which was kept at 80 °C. Data was collected at 1 scan/s through a 300–3000 m/z range in positive polarity and TOF resolution mode. For MS/MS a precursor ion carrying one modification from the most intense isotope cluster (+5 charge state) was selected, and CID fragmented with a 35–45 V collision energy ramp. A GluFib B solution was acquired in the reference function, and the correction was applied in-acquisition. Raw files were processed to.mzMLs using MSConvert, and the TopFD and TopMG modules of the TopPIC Suite were used for proteoform fragment ion assignment (Basharat et al. 2023).

## Results and discussion

### Selection of the reagents and MTs production

One of the most common methods used in metallothionein reactivity studies is monitoring the Zn(II) status following the protein’s reaction with reactive compounds. Oxidation or certain chemical modifications of cysteinyl sulfur significantly affect Zn(II) binding to cysteine residues and the overall zinc-binding site in the protein. Disulfides or alkylated sulfur atoms do not bind Zn(II), and their presence at native zinc sites can severely reduce binding affinity or cause Zn(II) dissociation. The extent of dissociation depends on the number of modified cysteine residues and may occur more readily in Zn(II)-buffered media (where free Zn(II) is controlled) (Krężel and Maret 2016). A straightforward way to study Zn(II) dissociation from Zn(II)-loaded MT is by using chromophoric probes that bind Zn(II) and exhibit changes in optical properties upon metal binding. Note that depending on the affinity of the chromophoric chelating probe, dissociated Zn(II) from native or weakened sites during modification can also be transferred. The most common zinc probes for studying Zn(II) dissociation from proteins and overall Zn(II) concentrations in samples are Zincon (2-carboxy-2′-hydroxy-5′-sulfoformazyl-benzene monosodium salt) and PAR (4-(2-pyridylazo)resorcinol) (Chen and Maret 2001; Chen et al. 2002; Kocyła et al. 2015; Kluska et al. 2018; Jacob et al. 1998). Zincon binds Zn(II) with micromolar affinity, forming a Zn-Zincon complex (1:1 stoichiometry), which absorbs light with a maximum at 618 nm (24,200 M^−1^·cm^−1^) (Kocyła et al. 2017). PAR binds Zn(II) with higher affinity in the subnanomolar range, forming a ZnH_x_(PAR)_2_ complex, which absorbs light with a maximum at 492 nm (71,500 M^−1^·cm^−1^) (Kocyła et al. 2015). Due to its higher molar absorption coefficient (approximately three times that of Zincon) and greater sensitivity, PAR was used in this study to investigate Zn(II) dissociation from MT upon its reaction with GOSC.

Six compounds were selected for their demonstrated reactivity toward macromolecules, including allicin (ALL), ajoene (AJO), and their metabolites such as S-allyl-L-cysteine (SAC), diallyl disulfide (DADS), diallyl sulfide (DAS), and allyl methyl sulfide (AMS) (Fig. 1b). ALL was synthesized according to established protocol (Fig. S1, S2), while the rest were purchased from commercial vendors (Albrecht et al. 2017). For metallothionein studies, four major human isoforms, MT1a, MT2, MT3, and MT4 were chosen (Table S1), though most experiments were conducted with MT2 due to its well-documented reactivity and Zn(II) binding affinity (Krężel and Maret 2021). All isoforms were produced as recombinant proteins according to established protocols (Table S2). Given their high reactivity and tendency to oxidize, the proteins were reduced after bacterial production, reconstituted with Zn(II) ions, and purified again. The final protein samples were analyzed for Zn(II) and thiolate content using PAR and DTNB (see Materials and Methods). All isoforms bound seven Zn(II) ions, and their identities were confirmed by mass spectrometry (Table S3).

### Effect of garlic compounds on Zn(II) dissociation from human MT2

**Fig. 2:**
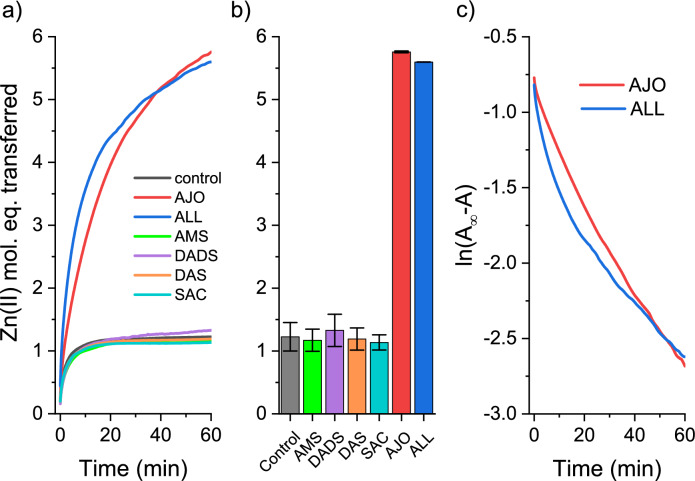
Zn(II) dissociation from Zn(II)-loaded MT2 by GOSC in the presence of PAR. **a** Kinetics of the reaction of 1 µM MT2 with 80 µM AJO, ALL, AMS, DADS, DAS, and SAC in the presence of 100 µM PAR in 50 mM HEPES-Na^+^, 100 mM NaCl, pH 7.4 at 25 °C; **b** comparison of the number of transferred Zn(II) mol. eq. at 60 min., depending on the garlic compounds used; **c** logarithmic replot of the allicin and ajoene kinetics given in **a**. Data are shown as means of n = 2 independent experiments ±SD

In the first approach, the reactivity of 1 µM MT2 was examined with GOSC at a concentration of 80 µM, which was four times higher than the cysteine residue content in MT2. First, 100 µM PAR was blanked with the appropriate agent at 492 nm, after which MT2 was added, and the reaction was monitored for 60 min (Fig. 2a). Absorbances were converted to Zn(II) molar equivalents transferred during the reaction using the molar absorption coefficient of ZnH_x_(PAR)_2_ (Kocyła et al. 2015). The recorded kinetics demonstrate that allicin is the most reactive in the initial phase of the reaction. Ajoene, which also shows high reactivity, is slower in the first phase but eventually (at 60 min) causes slightly higher Zn(II) dissociation from MT2 than allicin. This is best seen in Fig. 2b, which shows absorbances after 60 min of the reaction. Both reagents mobilize almost 6 Zn(II) mol. eq., indicating highly efficient protein modification under the applied conditions. The differences in the kinetics of ajoene and allicin likely result from different reaction orders. Ajoene reacts with MT2 according to first-order kinetics, while allicin is better characterized by second-order kinetics (Fig. 2c). Interestingly, AMS, DADS, DAS, and SAC show the same Zn(II) dissociation as the control, indicating no modification under the conditions used. The fact that 100 µM PAR alone can transfer ~1 Zn(II) mol. eq. is due to the presence of one weak binding site in MT2 with nanomolar affinity, which has been well-documented and characterized at the molecular level (Krężel and Maret 2007b; Singh et al. 2023; Mosna et al. 2023; Jiang et al. 1998). Therefore, the modification reactions of MTs by various compounds are influenced by the partial transfer of Zn(II) to PAR due to a competitive process. In this process, some Cys residues become more accessible to modifying compounds, accelerating the reaction rate.

**Fig. 3:**
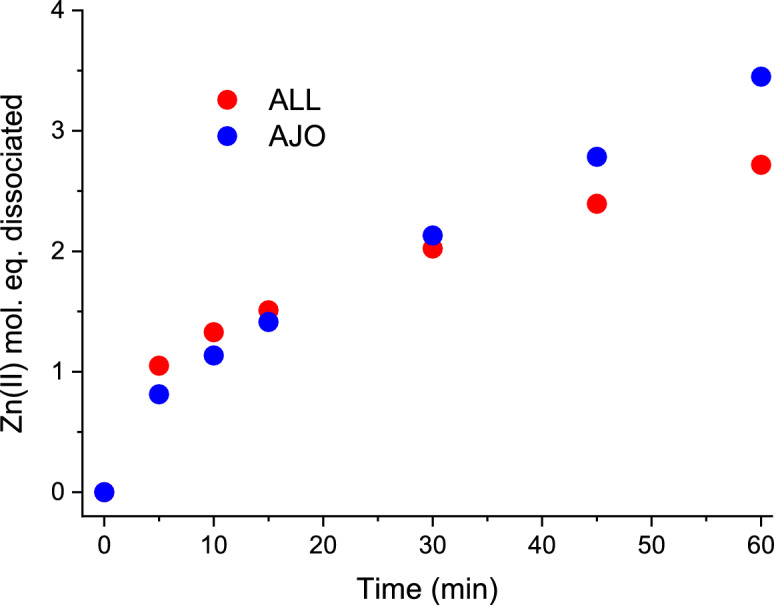
Zn(II) dissociation from Zn(II)-loaded metallothionein 2 (MT2) by allicin (ALL) and ajoene (AJO) without the presence of PAR. 1 µM MT2 was incubated with 80 µM ajoene (blue) or allicin (red) in a buffer containing 50 mM HEPES-Na^+^ and 100 mM NaCl at pH 7.4 at 25 °C. Samples were taken and tested for dissociated Zn(II) with 100 µM PAR just prior to measurement

**Fig. 4:**
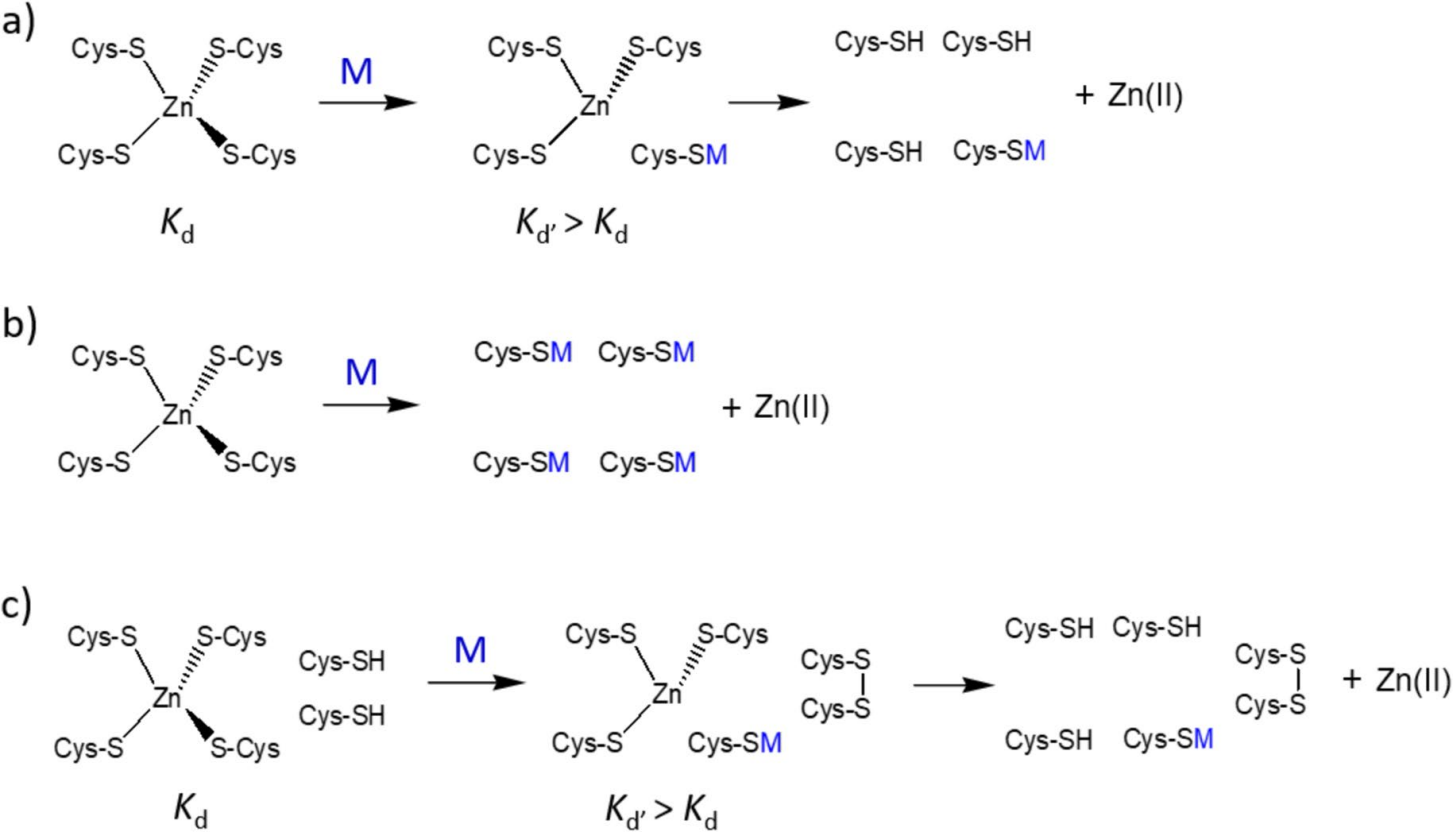
Proposed mechanisms of interaction between allicin and ajoene with MT lead to their modification and Zn(II) dissociation. **a** Partial S-thioallylation of the Zn(II) binding site results in the formation of a new site with lower Zn(II) affinity (*K*_d_’). **b** Full S-thioallylation of the Zn(II) binding site. **c** Partial S-thioallylation of the Zn(II) binding site combined with disulfide formation

To investigate how both GSOCs (allicin and ajoene) react directly with Zn(II)-loaded MT2, samples were prepared in the same way but without PAR and were incubated for the same period of time. To determine the mol. eq. of Zn(II) dissociated during the reaction, aliquots of the sample were taken during incubation and added to a cuvette with PAR just before measurement (Fig. 3). This approach significantly reduced the effect of protein and PAR competition for Zn(II). The results show that under the conditions used, fewer Zn(II) mol. eq. are mobilized. After 60 min, allicin causes the dissociation of ~2.5, while ajoene causes the dissociation of ~3 Zn(II) mol eq., respectively. The difference in kinetics between the two compounds remains visible and may be related to distinct reaction mechanisms, as proposed in Fig. 4. The lack of reactivity of compounds such as AMS, DAS, DADS, and SAC (Fig. 2a, b) was also confirmed during mass spectrometry measurements and will be discussed in the section on MT2 modification monitored by ESI–MS (see below).

**Fig. 5:**
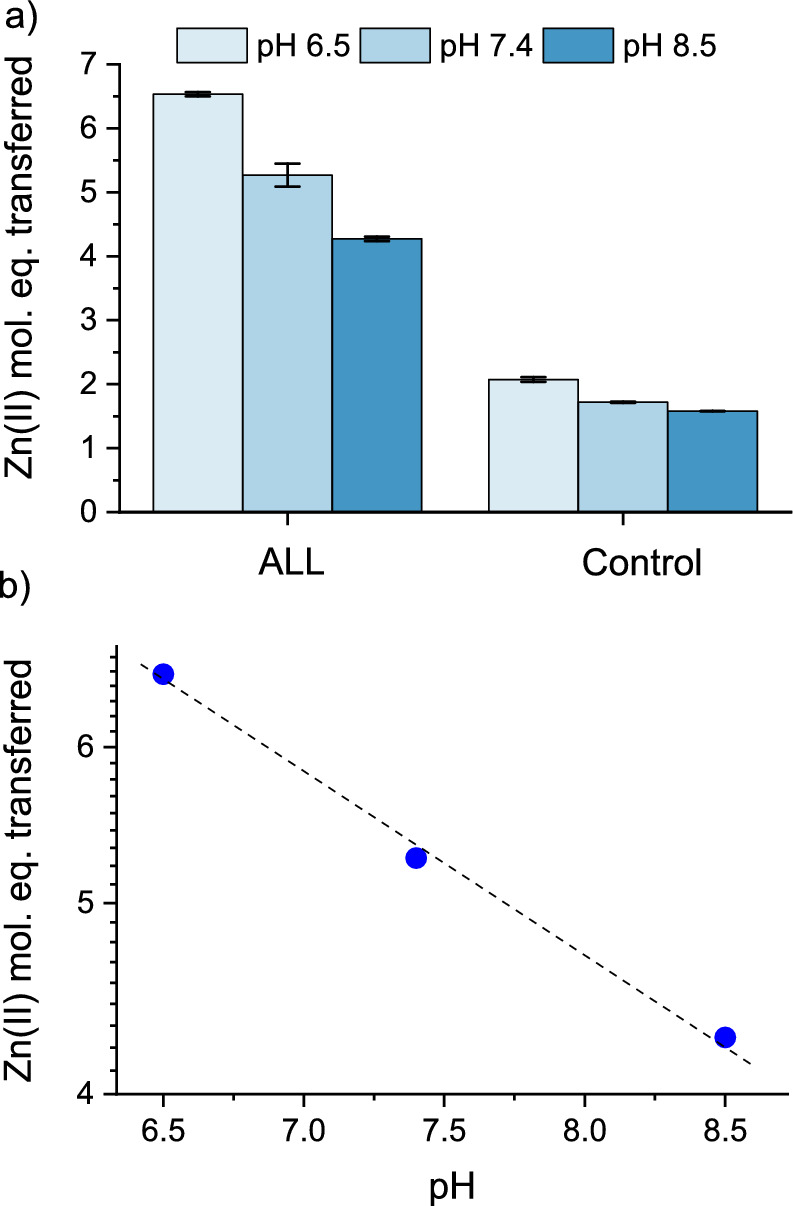
Zn(II) dissociation from Zn(II)-loaded metallothionein 2 (MT2) by 80 µM allicin (ALL) occurs in a pH-dependent manner. Molar equivalents of Zn(II) were transferred from 1 µM MT2 to 100 µM PAR in a buffer containing 50 mM HEPES-Na^+^ and 100 mM NaCl at pH 6.5, 7.4 and 8.5 after 1 h. Data are expressed as means of n = 2 independent experiments ±SD

The effect of buffer pH on the rate and efficiency of Zn(II) release during the reaction of MT2 with allicin was also investigated (Fig. 5). It was observed that both the rate and efficiency of the reaction decreased as the pH increased. This is attributed to the fact that at higher pH, MT2 binds Zn(II) more tightly due to differences in Cys thiol ionization. Consequently, the resulting binding sites at elevated pH exhibit lower reactivity compared to those in a more acidic environment (Krężel and Maret 2021). During the 1-h reactions of 1 µM MT2 with 80 µM allicin conducted at pH levels of 6.5, 7.4, and 8.5, the number of Zn(II) molar equivalents dissociated from MT2 was measured as follows: 6.5 at pH 6.5, 5.3 at pH 7.4, and 4.3 at pH 8.5.

**Fig. 6:**
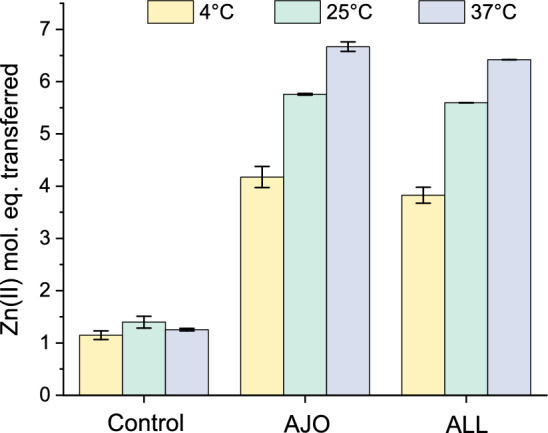
Zn(II) dissociation from Zn(II)-loaded metallothionein 2 (MT2) by 80 µM ajoene (AJO) and allicin (ALL) occurs in a temperature-dependent manner. Molar equivalents of Zn(II) were transferred from 1 µM MT2 to 100 µM PAR at temperatures ranging from 4 to 37 °C in a buffer containing 50 mM HEPES-Na^+^ and 100 mM NaCl at pH 7.4 after 1 h. Data are expressed as means of n = 2 independent experiments ±SD

**Fig. 7:**
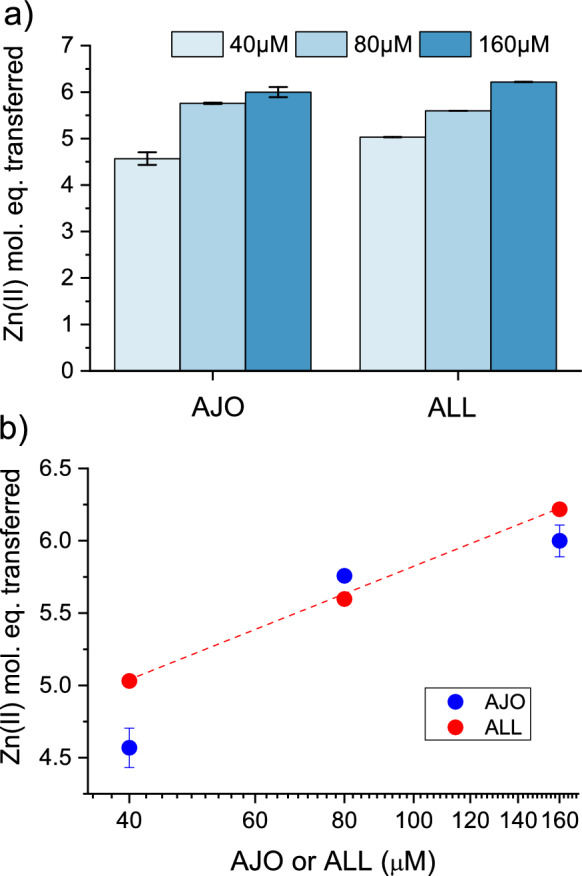
Zn(II) dissociation from metallothionein 2 (MT2) by ajoene (AJO) and allicin (ALL) occurs in a concentration-dependent manner. **A** Molar equivalents of Zn(II) transferred from 1 µM MT2 to 100 µM PAR were measured in the presence of 40–160 µM reactant in 50 mM HEPES-Na^+^ and 100 mM NaCl at pH 7.4 at 25 °C after 1 h; **B** the same Zn(II) transfer is presented on a logarithmic scale of the reactant concentration. The red dashed line indicates a linear trend for ALL. Data are expressed as means of n = 2 independent experiments ±SD

In the next step of the study, the reaction of allicin and ajoene with MT2 was also examined as a function of changing temperature. An analogous reaction to that presented in Fig. 2 was performed at 4 °C, resulting in the mobilization of 4.2 and 3.9 Zn(II) mol. eq. for ajoene and allicin, respectively (Fig. 6). Reactions performed at 37 °C increased the number of Zn(II) mol. eq. dissociated from the protein to 6.5 and 6.2 Zn(II) mol eq. for ajoene and allicin, respectively. At all tested temperatures, the reactivity of ajoene was slightly more efficient than that of allicin. Since the reactivity of the two reagents differed slightly, we examined in the next step how Zn(II) dissociation from MT2 behaves at various reagent concentrations, ranging from 40 to 160 µM. Figure 7b demonstrates a more linear increase in reaction efficiency for allicin than for ajoene, where reactivity at 40 µM was more efficient than for allicin. This change in the order of reactivity at lower concentrations of the reactive reagent is likely due to differences in the kinetic order of both reagents and different mechanisms.

### Comparison of allicin’s effect on MT1-MT4 isoforms

**Fig. 8:**
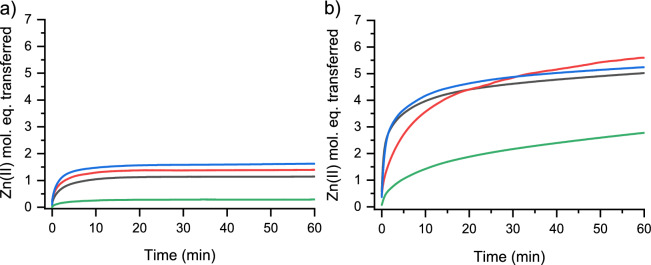
Zn(II) dissociation from human MT1-MT4 isoforms by allicin. The time dependence of Zn(II) transfer from 1 µM MT1a (black), MT2 (red), MT3 (blue), and MT4 (green) to 200 µM PAR is presented in **a** the absence and **b** the presence of 80 µM allicin in 50 mM HEPES-Na^+^, 100 mM NaCl, pH 7.4 at 25 °C. Data are shown as means of n = 2 independent experiments

In the final stage of the reactivity study of GSOCs, we tested how allicin (80 µM) reacts with various Zn(II)-loaded metallothionein isoforms (MT1a, MT2, MT3, and MT4). In all kinetics, the same concentration of MT isoform was used, and Zn(II) transfer was monitored as described above in the presence of 100 µM PAR. The kinetics of the reactions are presented in Fig. 8 and show substantial differences in the course of the reaction. MT1a and MT3 react very rapidly in the first phase of the reaction, reaching approximately 4 Zn(II) mol. eq. dissociation, after which the reactions slow down. Their courses are almost identical, indicating mobilization of 5 and 5.2 Zn(II) mol. eq. after 60 min, respectively. The reactivity of MT2 and MT4, although differing in terms of efficiency, demonstrates a similar course. MT4 is the least reactive isoform. Its treatment with allicin causes dissociation of only 2.8 Zn(II) mol. eq. after 60 min. The low reactivity of MT4 compared to other isoforms was recently observed in arsenical labeling and is attributed to the more compact structure of this isoform, which is due to the presence of hydrophobic residues (Pomorski et al. 2025).

### CD-monitored Zn(II) dissociation from MT2 upon S-thioallylation

**Fig. 9:**
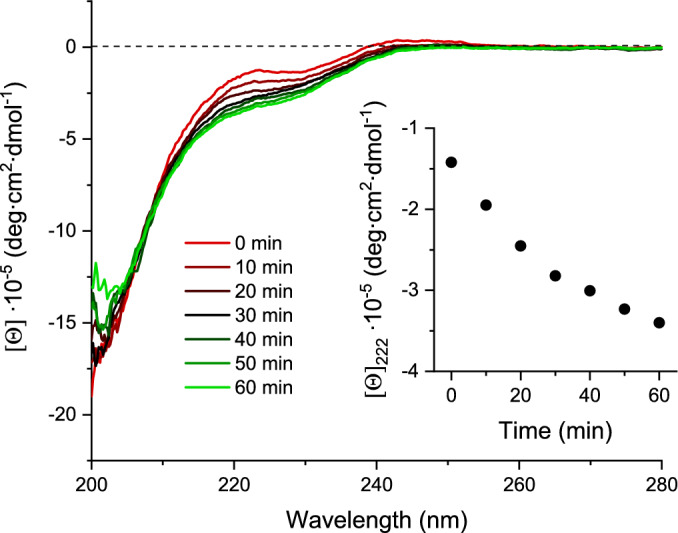
CD-monitored reaction between 800 µM allicin and 10 µM MT2 in 20 mM Tris–HCl, 100 mM NaClO_4_, pH 7.4. Inset indicates molar ellipticities changes at selected 222 nm as a function of time reaction

Circular dichroism (CD) measurements in the 180–240 nm range are typically used to determine the percentage and type of secondary structure in the analyzed protein molecule. However, in the case of metallothioneins, this is hardly possible due to the lack of secondary structures (the 3D architecture of the protein is based on the clusters formed) and strong optically active, metal-ion-induced transitions, such as ligand-to-metal charge transfer (LMCT) (Freisinger and Vašák 2013). Nevertheless, such intense transitions can be used to investigate the stoichiometry of metal ions binding or to monitor the dissociation of these ions. To examine the effect of S-thioallylation on the conformation of metallothionein, 10 µM MT2 (5 µM in case of ajoene) was incubated with 800 µM allicin or 400 µM ajoene, and CD spectra were recorded every 10 min (Fig. 9 and Fig. S3). The change in molar ellipticity observed in the range from ~210 to ~250 nm, with a maximal change at 222 nm, clearly indicates that the MT2 conformation, which is hardly observable in CD spectra, changes during the reaction due to Zn(II) dissociation and Cys residue alkylation. Moreover, the time-dependent conformational change (inset of Fig. 9) observed in this study is comparable to the Zn(II) dissociation observed using the chromogenic chelator PAR (Fig. 2a), despite differences in reactant concentration. Importantly, the observed effect cannot be explained (exclusively) as a structure change but rather as a consequence of Zn(II) dissociation due to S-thioallylation.

### S-thioallylation of MT2 is reversible

To investigate the ability of MT2 to rebind Zn(II) after the reduction of Cys residues by a reducing agent, the Zn(II)-specific chromogenic chelator Zincon was used. This agent has a lower affinity for Zn(II) than the previously used chelator PAR. The use of Zincon was intended to reduce competition for Zn(II) between the weaker Zn(II) binding sites in MT2 and the chelator. First, MT2 was treated with allicin, as in the previous measurements with PAR, and after 1 h, a reducing agent was added (Fig. S4a). Only in the case of TCEP was the observed effect attributable to the rebinding of Zn(II) to MT2, as the same effect was also observed in control samples for GSH and DTT (Fig. S4b). This is related to the higher apparent -log*K*_d_ values of Zn(II) with the used reducing agent, which are 5.2 for Zn(II)-GSH, 8.32 for Zn(II)-DTT, and 3.2 for Zn(II)-TCEP (Krężel et al. 2003a, Adamczyk et al. 2015, Krężel et al. 2003b). The results of this experiment reveal that under cellular conditions, modified metallothionein can indeed be reduced. It means that, after undergoing the S-thioallylation reaction, the protein remains active. Instead, it can re-enter the protein pool as a fully functional participant in maintaining Zn(II) homeostasis. Consequently, the effect of allicin is temporary rather than permanent, which offers health benefits while minimizing side effects.

### MT2 modification monitored by ESI–MS

**Fig. 10:**
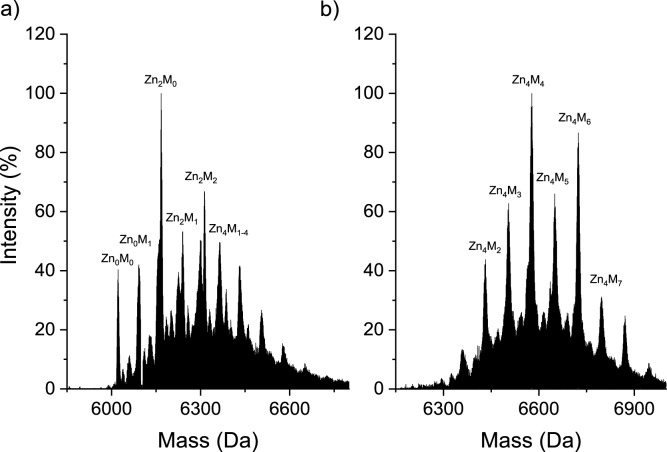
ESI–MS spectra of 40 µM Zn(II)-loaded MT2 in a molar ratio of 1:80 with **a** ajoene and **b** allicin, recorded after 1 h of reaction at 25 °C

In the second part of the study, mass spectrometry was used to monitor MT2 modification and the dissociation of Zn(II) from fully Zn(II)-loaded protein. It was shown that in the 1-h reaction of saturated Zn_7_MT2 with ajoene or allicin, variously saturated, modified, and partially oxidized complexes are formed (Fig. 10). In the case of ajoene, we observed a greater diversity of complexes in terms of Zn(II) saturation, such as Zn_0_M_0-1_MT2, Zn_1-3_M_0-2_MT2, and Zn_4_M_1-4_MT2 (where M indicates modification), compared to the complexes obtained from the reaction of MT2 with allicin, such as Zn_4_M_1-8_MT2. All identified complexes are presented in Table S4. These results are similar to spectrophotometric measurements of the allicin + MT2 reaction without PAR (Fig. 3), where we observed 2.5–3.5 molar equivalents of dissociated Zn(II) at the same reaction time. However, in the spectroscopic study, only the average effect of Zn(II) dissociation is observed. Mass spectrometry is a high-resolution method that differentiates modification, oxidation, and Zn(II) dissociation. With that, we observed that the oxidation effect is more pronounced in the case of ajoene than in allicin, which may be related to the different reaction mechanisms of these compounds, as also noted in the differing kinetic rates observed in spectroscopic measurements. Taking spectrophotometric and mass spectrometry measurements into consideration, it seems that the mechanism of ajoene corresponds to a pathway dominated by the formation of intramolecular disulfide bonds (Fig. 4c). In the case of allicine, however, the path is dominated by the modification of Cys (Fig. 4a-b). S-thioallylation of Cys residues in the Zn(II) binding site results in the formation of a new site with lower Zn(II) affinity (*K*_d_’), leading to metal ion dissociation. Decreasing Zn(II) binding site affinity may also be caused by the formation of disulfide bridges in MT2, which may accompany S-thioallylation. For garlic organosulfur compounds, AMS, DADS, DAS, and SAC modification of MT2 was not observed (Tab. S4, Fig. S5). The lack of reactivity of GOSC such as AMS, DAS, DADS, and SAC is related to the absence of a sufficiently strong nucleophilic atom. The literature describes the relationship between the activity of these compounds and the number of sulfur atoms in the polysulfide chain (Kaschula and Hunter 2016). The more sulfur atoms that are adjacent to each other, the greater the activity exhibited by the compound. This may be related to the dissociation energy of the central bond; the more sulfur atoms present in the chain, the lower this energy, making the molecule more reactive. Moreover, ajoene and allicin have a polarized bond between oxygen and one of the sulfur atoms, making these molecules more reactive compared to a simple disulfide toward nucleophilic thiol groups (Gruhlke et al. 2019). However, it should be mentioned that the most reactive compound causing oxidative Zn(II) dissociation from rabbit MTs (both MT1 and MT2) is ebselen. It reacts more rapidly than allicin and ajoene with all cysteine residues, causing the dissociation of seven Zn(II) ions due to the presence of highly reactive selenium atoms and the formation of disulfide bonds (Jacob et al. 1998).

**Fig. 11:**
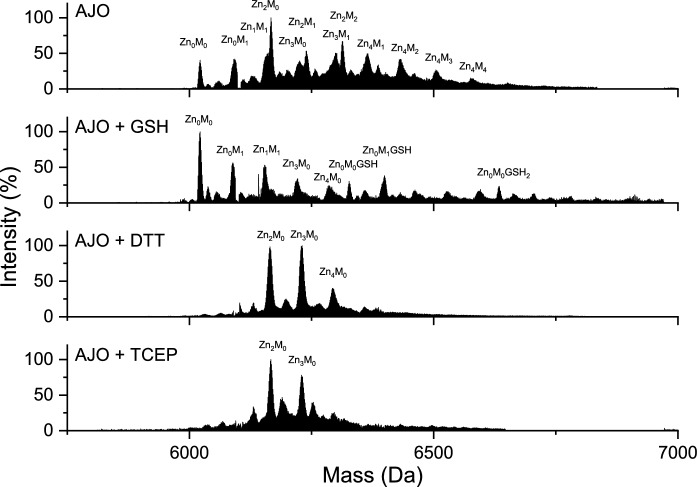
ESI–MS spectra of 40 µM MT2 with ajoene in a molar ratio of 1:80, along with the addition of GSH, DTT, and TCEP in a molar ratio of 1:100, were recorded after 1 h of reaction at 25 °C. Zn_X_M_Y_GSH_Z_ denotes complexes of MT2 with GSH with various numbers of modifications

**Fig. 12:**
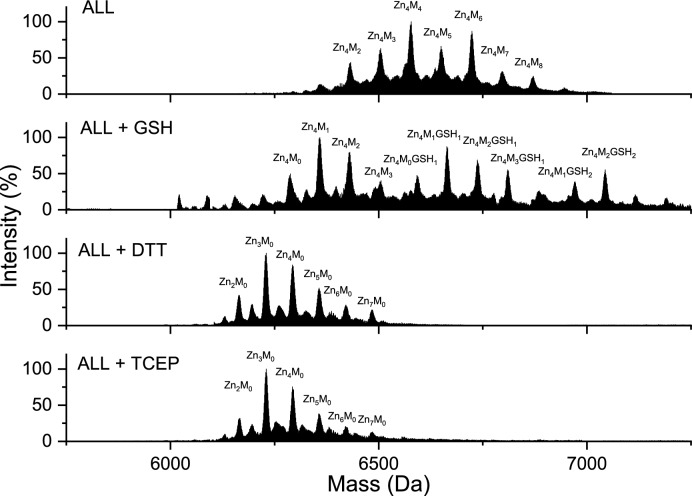
ESI–MS spectra of 40 µM MT2 with allicin in a molar ratio of 1:80, along with the addition of GSH, DTT, and TCEP in a molar ratio of 1:100, were recorded after 1 h of reaction at 25 °C. Zn_X_M_Y_GSH_Z_ denotes complexes of MT2 with GSH with various numbers of modifications

Previous studies indicate that the modification of cysteine residues in the S-thioallylation reaction is reversible upon the application of the reducing agents DTT or GSH (Rabinkov et al. 1998). Papain and alcohol dehydrogenases regained enzymatic activity after the use of reducing agents, as the cysteine residues essential for enzymatic activity were reduced (Rabinkov et al. 1998). In this study, a fivefold excess of reducing agents with different reduction potentials, GSH, DTT, and TCEP, was applied relative to the cysteine residues. The reversibility of the reaction was observed in samples treated with both tested GOSC (Figs. 11, 12). The 15-min incubation with GSH resulted in partial removal of the modification, whereas incubation with DTT and TCEP, it was completely reversible. However, in all samples with the addition of GSH, including the control, one or more GSH molecules were found to be bound to MT2, marked as complexes Zn_X_M_Y_GSH_Z_, where X denotes the number of bound Zn(II) ions, Y is the number of modifications, and Z denotes the number of attached glutathione molecules (Figs. 11, 12, Fig. S6, Table S5, S6). The complexes with ajoene Zn_0_M_0-1_MT2_ox_GSH_1-2_ and with allicin Zn_4_M_1-3_MT2_ox_GSH_1-2_ were detected. This confirms earlier reports that such interactions between GSH and metallothioneins occur, highlighting the relationship between these two compounds that depends on the cell’s redox state (Zalewska et al. 2014). Glutathione can act protectively on metallothionein and reduce its cysteine residues, but when in its oxidized form (GSSG), it can promote their oxidation (Maret 1994). The lack of fully saturated metallothionein after adding the reducing agent is due to the method employed. When MT2 reacted with AJO or ALL, the buffer was exchanged, effectively removing the dissociated Zn(II) from the sample. Consequently, when the reducing agent was added, Zn(II) re-binding did not take place.

### Identification of Cys modification by ajoene and allicin in Zn_7_MT2

For this experiment, aimed at identifying the most GOSC-reactive site in MT2, a top-down protein MS approach was chosen. Contrary to the bottom-up approach, where peptides are first enzymatically generated from a protein molecule, in a top-down protein MS analysis, an intact protein molecule is ionized and then fragmented using collision energy, with resulting fragments being identified (Peris-Díaz et al. 2023; Peris-Díaz et al. 2024). In our case, the main advantage of the top-down approach over the bottom-up approach was the elimination of nonspecific modifications that may occur on MT2 tryptic peptides in the absence of a reducing agent (i.e. cysteine oxidation), which we established as a prerequisite of MT2 modification by GOSC (Figs. 11, 12) (Bernard 1991). Furthermore, the top-down method allows for the selection of a precursor ion derived from the entire ionized protein molecule, including any modification. Hence the possibility of missing a modification site due to its presence in a weakly ionizing peptide is avoided.

**Fig. 13:**
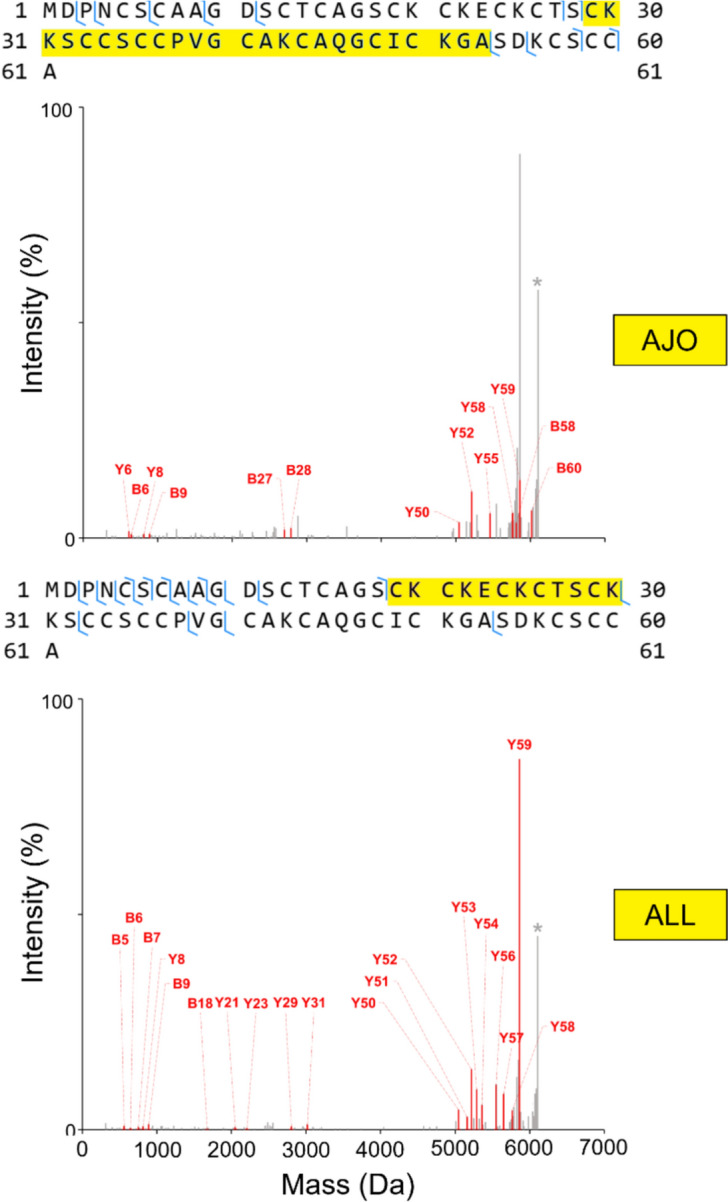
Top-down analysis of MT2 singly modified by ajoene (AJO) or allicin (ALL). Light blue marks on MT2 sequences indicate observed BY-type bond cleavages, and in yellow, the GOSC-modified regions are highlighted. On the fragment mass distribution panes, the red sticks indicate matched BY ions for each case, while the gray sticks indicate unmatched ions. The modified MT2 precursor ions are annotated with an asterisk. In both cases, the best-scoring PrSMs are presented, and the mass error tolerance used was ±25 ppm

To identify the most reactive site in MT2, lower concentrations of ajoene and allicin were used, ensuring a 1:1 molar ratio of these compounds to the cysteine residues present. It allowed for the introduction of a single modification per MT2 molecule. The reaction was conducted in 50 mM ammonium acetate at pH 7.4 for 15 min. The selected top-down method allowed us to narrow down the modification sites to regions containing several cysteine residues. Attempts to generate a higher number of bond cleavages for more discrete spectra by applying higher collision energy resulted in a loss of an interpretable fragmentation pattern. In the case of both studied modifications, Protein-Spectrum-Matches (PrSMs) with an E-value below 10⁻^5^ were obtained (Table S7, S8). The E-value parameter evaluates spectra to proteoform sequence match quality, and the E-value ≤0.00001 is the most typically used cut-off threshold. The best-matching PrSMs for ajoen and allicin had E-values of 1.12 · 10^–7^ and 1.12 · 10^–8^, respectively, and are shown in Fig. 13 (top panel). Given this data, it can be concluded that the regions prone to modification are located near the KKS linker on either the N-terminal or C-terminal side of the protein, depending on the modifier studied. For the sample with ajoene, the determined PrSM’s modification mass was +68.9884 Da, which is lower than the expected value (+71.9995 Da) but could be due to the oxidation of cysteine residues, as observed during previous mass spectrometry measurements (Fig. 10, Table S4). The ajoen-modified region comprises residues 29–53, limiting this modification to either of the nine cysteines present therein. These being Cys29, Cys33–34, Cys36–37, Cys41, Cys44, Cys48 or Cys50. In the case of the sample with allicin, the obtained PrSM showed an expected modification of +71.9995 Da (Fig. 13 bottom panel). In accordance with the more predictive nature of the allicin modification, a much better fragment sequence coverage can be observed for the allicin sample compared to the ajoene sample. Allicin seems to modify MT2 within residues 19–30, indicating either Cys19, Cys21, Cys24, Cys26, or Cys29 as being this compound’s reactive site. Based on the results, it can be concluded that the GOSC modification of MT2 does not occur exclusively on a single site, as the identified PrSMs’ modified sequences do not overlap.

Based on the X-ray structure of rat MT2 (PDBID: 4MT2), which is very similar to human MT2, identified modified regions appear to be more exposed and accessible to the modifier (Robbins et al. 1991). Previous studies on the effects of drugs such as melphalan and chlorambucil, using the bottom-up mass spectrometry method, have shown that these compounds modify Cys33, the first cysteine from the C-terminal side of the KKS linker, and Cys48 (Yu et al. 1995; Zaia et al. 1996). It is worth noting that these studies examined metallothioneins saturated with Cd(II) ions, which may also influence the specificity of cysteine residue modifications due to the larger atomic radius of Cd(II) compared to Zn(II), higher affinity, and different folding mechanisms (Krężel and Maret 2021; Peris-Díaz et al. 2021). Additionally, the size and nature of the modifying molecule may play a role. Both melphalan and chlorambucil are larger molecules than most of the compounds studied in this work, and they possess aromatic rings in their chains, which increases their hydrophobicity. It is also worth mentioning that in our study, a few other PrSMs were identified with an E-value below 10^–5^ in the case of allicin, as well as ajoene (Table S7, S8). This may indicate that the MT2 population with a single modification is not homogeneous and that several differently modified molecules are present.

The modification of MT2 by the compounds studied in this work, and consequently the dissociation of Zn(II) ions, complement the mechanisms of action of ajoene and allicin. This is particularly relevant, as cellular studies have demonstrated that allicin induces the release of Zn(II) ions in EL-4 cells and macrophages (Gruhlke et al. 2019; Haase et al. 2012). The literature presents highly variable values regarding the percentage of Zn(II) ions bound by metallothioneins within a cell (Coyle et al. 2002; Kimura and Kambe 2016; Jarosz et al. 2017; Wang et al. 2018). This depends not only on the organism but also on the cell type and investigating method. It has been found that in the hepatocytes of marine mammals, metallothioneins are responsible for binding up to 51% of Zn(II), whereas in the kidneys, this value is approximately 14% (Das et al. 2006). In contrast, in the HT-29 colon cancer cell line, it was determined that metallothioneins can bind up to 10% of Zn(II) (Krężel and Maret 2006). Nevertheless, metallothioneins constitute an important reservoir of Zn(II) ions in organisms, so modifications of these proteins that lead to Zn(II) dissociation may have health-promoting effects (Jarosz et al. 2017). It has been demonstrated that Zn(II) ions inhibit the activation of the NF-κB factor, which is responsible for the expression of pro-inflammatory molecules such as IL-1β, IL-6, IL-8, and TNF-α, as well as pro-inflammatory enzymes like cyclooxygenases and nitric oxide synthase (Prasad 2014). Additionally, Zn(II) ions regulate the upregulation of Nrf2 and inhibit NADPH oxidase, which participates in the formation of reactive oxygen species. Zn(II) ions are also essential for T lymphocyte activation and, thus, for the immune response (Maywald et al. 2017). A wide range of zinc-dependent enzymes have been identified, including superoxide dismutase and alcohol dehydrogenase. Several studies have indicated the positive effects of ajoene and allicin, which may be linked to Zn(II) ions. In a mouse model of alcoholic fatty liver disease, it was demonstrated that the presence of allicin in the diet resulted in a decrease in TNFα, IL-1β, and IL-6 levels while increasing alcohol dehydrogenase activity (Panyod et al. 2016). Furthermore, the use of allicin and ajoene in macrophage cultures suppressed the expression of pro-inflammatory IL-1β, IL-6, and IL-12β (Hitchcock et al. 2021). The S-thioallylation reaction can lead to the dissociation of Zn(II) from Zn(II)-dependent enzymes or repressor factors within oxidative stress pathways, playing a significant role in combating infections. This process can work in two ways: it can inactivate crucial enzymes essential for pathogen survival, leading to their death, or it can trigger protective mechanisms that allow pathogens to survive (Müller et al. 2016; Borlinghaus et al. 2021). It is important to remember that this reaction specifically targets cysteine thiols. Meanwhile, Zn(II) ions can also be coordinated by histidine, aspartate, and glutamate residues (Krężel and Maret 2016).

In this study, we explored the dissociation of Zn(II) ions from metallothioneins (MTs) induced by garlic-derived compounds, allicin, and ajoene. While our findings provided significant insights into this interaction, it is crucial to recognize that MTs are not solely limited to Zn(II) binding. They also have the capacity to bind other metal ions, including toxic heavy metals such as cadmium (Cd(II)), lead (Pb(II)), and mercury (Hg(II)). This opens up a vital avenue for future research: determining whether allicin and ajoene can induce the dissociation of these harmful metal ions from metallothioneins in a similar manner. Understanding this interaction could provide a deeper insight into the therapeutic potential of garlic compounds in alleviating heavy metal toxicity. For instance, previous studies have demonstrated that allicin administration in rats exposed to toxic doses of Pb(II) alleviated learning and memory deficits (Cai et al. 2019). Furthermore, allicin has been shown to reduce Pb(II) concentrations in blood and tissues while also decreasing Zn(II) levels in the liver of mice (Aslani et al. 2011). The therapeutic effect of allicin in cases of lead poisoning is attributed to its dual capacity: it not only mobilizes lead from tissues, facilitating its elimination from the body, but also helps mitigate the oxidative stress that contributes to lead ion toxicity. This mechanism underscores the importance of further investigation into the protective effects of garlic-derived compounds against various heavy metals.

## Conclusion

Our research shows that among the active organosulfur compounds derived from garlic studied, only ajoene and allicin cause the dissociation of Zn(II) ions from human metallothioneins (MT1-MT4) and induce S-thioallylation of cysteine residues in MTs. The effects of the dissociation of Zn(II) and modification of metallothionein may be linked to the health-promoting properties of these studied compounds. However, at least at the cellular level, direct evidence is needed for the relationship between the action of the studied active compounds and metallothioneins, the dissociation of Zn(II) ions, and their impact on specific signaling pathways.

## Supplementary Information

Below is the link to the electronic supplementary material.

Supplementary file 1 (DOCX 1987 KB)

## Data Availability

No datasets were generated or analysed during the current study.
