# Supplementary material for: Modification of human metallothioneins by garlic organosulfur compounds, allicin and ajoene: direct effect on zinc homeostasis with relevance to immune regulation
Source: Biometals. 2025 Jul 30;38(5):1513–33. doi: 10.1007/s10534-025-00716-3 (PMC12507958; doi:10.1007/s10534-025-00716-3)
Supplement: Supplementary file 1 — (DOCX 1987 KB) [file 10534_2025_716_MOESM1_ESM.docx]

*Supplementary data*

**Modification of human metallothioneins by garlic organosulfur compounds, allicin and ajoene: Direct effect on zinc homeostasis with relevance to immune regulation**

Karolina Mosna^1^, Alicja Orzeł^1^, Michał Tracz^2^, Sylwia Wu^1^ and Artur Krężel^1,2*^

*^1^Department of Chemical Biology, Faculty of Biotechnology, University of Wrocław, Joliot-Curie 14a, 50-383 Wrocław, Poland*

*^2^Laboratory of Protein Mass Spectrometry, Faculty of Biotechnology, University of Wroclaw,
Joliot-Curie 14a, 50-383 Wrocław, Poland*

**Keywords**: S-thioallylation, labile zinc, protein modification, zinc homeostasis, reactive species

* To whom correspondence should be addressed, E-mail: [artur.krezel@uwr.edu.pl](mailto:artur.krezel@uwr.edu.pl)


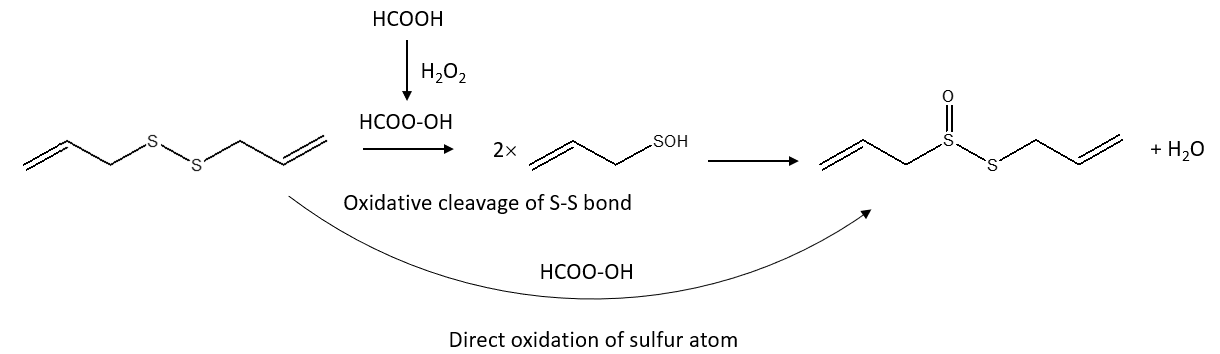


**Figure S1.** Scheme of allicin synthesis from diallyl disulphide (DADS) using formic according to Albrecht at al. 2017.

#
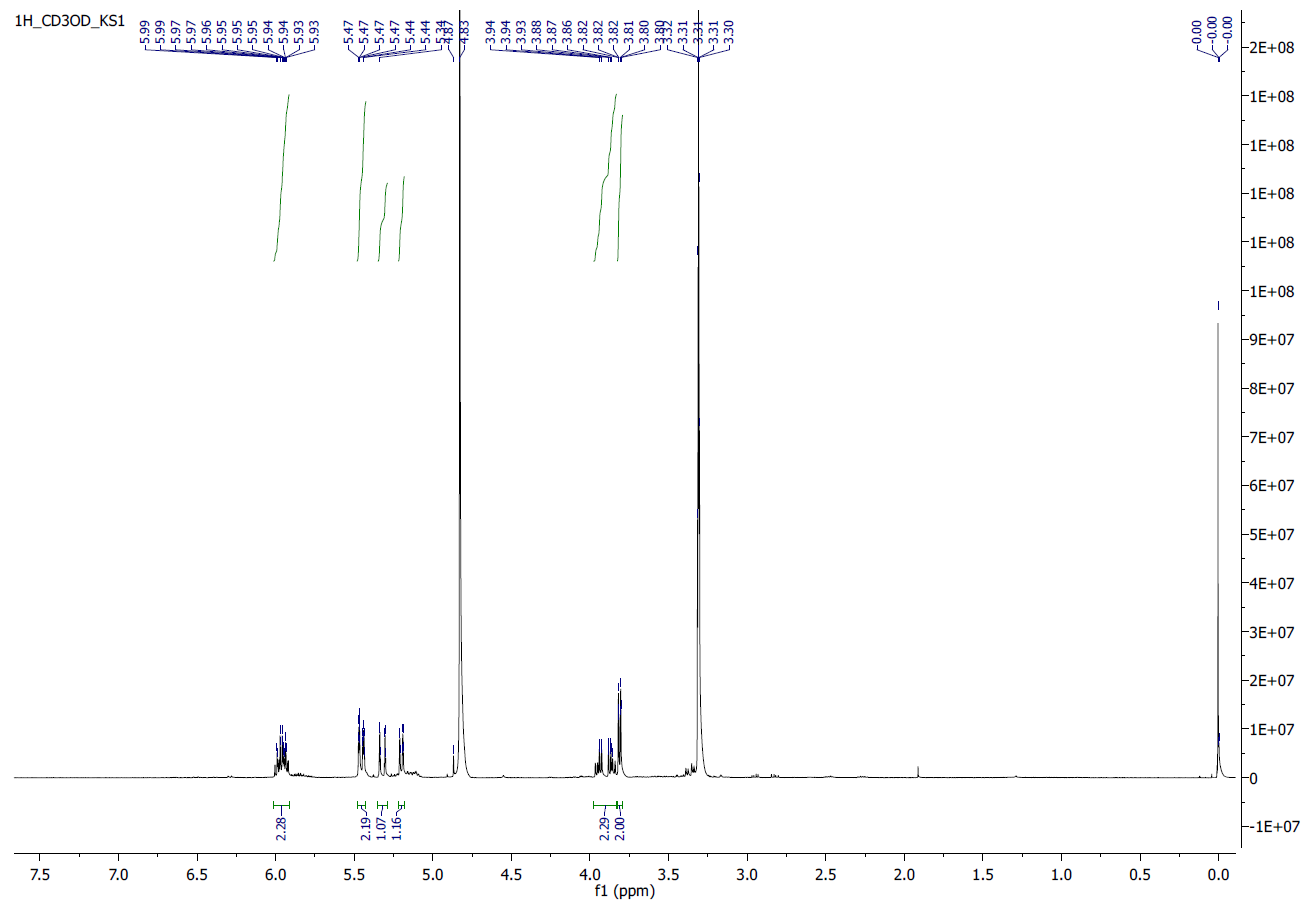


# Figure S2. ^1^H NMR spectrum of synthesized allicin recorded in CD_3_OD.

# Table S1. The sequences of human metallothionein isoforms used for experiments.

| MT isoform | Amino acid sequence |
| --- | --- |
| MT1a | MDPNCSCATGGSCTCTGSCKCKECKCTSCKKSCCSCCPMSCAKCAQGCICKGASEKCSCCA |
| MT2 | MDPNCSCAAGDSCTCAGSCKCKECKCTSCKKSCCSCCPVGCAKCAQGCICKGASDKCSCCA |
| MT3 | MDPETCPCPSGGSCTCADSCKCEGCKCTSCKKSCCSCCPAECEKCAKDCVCKGGEAAEAEAEKCSCCQ |
| MT4 | MDPRECVCMSGGICMCGDNCKCTTCNCKTCRKSCCPCCPPGCAKCARGCICKGGSDKCSCCP |

# Table S2. Molecular masses of apo-MT isoforms obtained in this study.

| MT isoform | Found masses (Da) | Calculated masses (Da) |
| --- | --- | --- |
| MT1a | 6122.9318 | 6120.3405 |
| MT2 | 6041.2158 | **6042.2072** |
| MT3 | 6924.4875 | **6927.0032** |
| MT4 | 6418.4676 | **6418.7964** |

# Table S3. Molecular masses of Zn(II)-loaded MT isoforms obtained in this study.

| MT isoform | Found masses (Da) | Calculated masses (Da) |
| --- | --- | --- |
| MT1a | 6563.6428 | 6562.7141 |
| MT2 | 6484.4366 | 6484.7002 |
| MT3 | 7369.4125 | 7368.9925 |
| MT4 | 6861.8604 | 6860.8981 |

#

# Figure S3. CD-monitored reaction between 400 µM ajoene and 5 µM MT2 in 20 mM Tris-HCl, 100 mM NaClO_4_, pH 7.4. Inset indicates molar ellipticities changes at selected 222 nm as a function of time reaction.

# Figure S4. Zn(II) dissociation from Zn(II)-loaded metallothionein 2 (MT2) by 80 µM allicin to 100 µM Zincon and reversible transfer after the addition of 1 mM TCEP. The reaction was performed in a buffer containing 50 mM HEPES-Na^+^ and 100 mM NaCl at pH 7.4. b) Control reactions contain 3 eq. mol Zn(II) saturated Zincon, 80 µM allicin, and 1 mM GSH, DTT, or TCEP in a buffer containing 50 mM HEPES-Na^+^ and 100 mM NaCl at pH 7.4.

# Table S4. Identified complexes in ESI-MS for the 1h reaction of 40 µM MT2 with garlic compounds in molar ratio 1:80 in 25°C. Zn_x_MT_ox_ and Zn_X_M_Y_MT denote oxidized metallothionein complex and modified metallotionein complex, respectively. M and y stand for modification and the number of conjugated residues of reactive species.

| Reagent | Identified complex | Found masses (Da) | Calculated masses (Da) |
| --- | --- | --- | --- |
| Control | Zn_7_MT  Zn_6_MT | 6484.4366  6419.2102 | 6484.7002  6420.7710 |
| Ajoen | Zn_0_M_0_MT_ox_  Zn_0_M_1_MT_ox_  Zn_1_M_1_MT_ox_  Zn_2_M_0_MT  Zn_2_M_0_MT·MeOH  Zn_2_M_1_MT  Zn_3_M_1_MT_ox_  Zn_2_M_2_MT  Zn_4_M_1_MT  Zn_4_M_2_MT_ox_  Zn_4_M_3_MT_ox_  Zn_4_M_4_MT_ox_ | 6021.8966  6091.8647  6158.8758  6166.8977  6201.8674  6238.8798  6298.7840  6312.8881  6363.7162  6431.6765  6504.6808  6575.6817 | 6022.0791  6092.0551  6160.0239  6165.0544  6198.0879  6237.0544  6300.9836  6309.0544  6364.9127  6436.9127  6508.9127  6580.9127 |
| Allicin | Zn_4_M_1_MT_ox_  Zn_4_M_2_MT_ox_  Zn_4_M_3_MT_ox_  Zn_4_M_4_MT_ox_  Zn_4_M_5_MT_ox_  Zn_4_M_6_MT  Zn_4_M_7_MT  Zn_4_M_8_MT | 6359.7864  6432.7753  6504.7649  6577.7704  6649.7813  6723.7967  6796.7930  6870.8044 | 6359.8815  6432.8815  6504.8815  6577.8893  6649.8893  6724.9127  6796.9127  6868.9127 |
| AMS | Zn_7_MT  Zn_6_MT | 6484.4437  6419.2735 | 6484.7002  6420.7710 |
| DADS | Zn_7_MT  Zn_6_MT_ox_ | 6484.4868  6417.5152 | 6484.7002  6420.7710 |
| DAS | Zn_7_MT  Zn_6_MT_ox_ | 6484.5344  6415.7984 | 6484.7002  6420.7710 |
| SAC | Zn_7_MT  Zn_6_MT_ox_ | 6484.2759  6417.4122 | 6484.7002  6420.7710 |

#

# Figure S5. ESI-MS spectra of 40 µM MT2 (noted as Zn_7_MT2) a) without any reagent (control) and incubated for 1 h with b) AMS, c) DADS, d) DAS, and e) SAC in molar ratio 1:80, 25°C.

#

# Figure S6. ESI-MS spectra of 40 µM MT2 with the addition of GSH, DTT and TCEP in ratio 1:100. Zn_7_MT·GSH_x_ are complexes of fully Zn(II)-loaded MT2 with GSH. Zn_7_MT*Na^+^ are complexes of fully Zn(II)-loaded MT2 with Na^+^ adducts.

# Table S5. Identified complexes in ESI Mass Spectra of control reaction 40 µM MT2 with reducers GSH, DTT and TCEP in ratio 1:100.

| **Reagent** | **Identified complex** | **Found masses (Da)** | **Calculated masses (Da)** |
| --- | --- | --- | --- |
| MT2 | Zn_7_MT | 6484.4975 | 6484.7002 |
| MT2 + GSH | Zn_7_MT  Zn_0_MTGSH_1_  Zn_0_MTGSH_2_  Zn_0_MTGSH_3_ | 6485.4873  6791.5809  7099.6651  7405.7406 | 6484.7002  6791.7840  7098.8678  7405.9516 |
| MT2 + DTT | Zn_7_MT | 6485.4833 | 6484.7002 |
| MT2 + TCEP | Zn_7_MT  Zn_7_MT*Na^+^  Zn_7_MT*Na_2_^+^  Zn_7_MT*Na_3_^+^  Zn_7_MT*Na_4_^+^  Zn_7_MT*Na_5_^+^  Zn_7_MT*Na_6_^+^ | 6485.4733  6508.4613  6530.4467  6553.4187  6575.4375  6595.4030  6618.4043 | 6484.7002  6507.6894  6530.6786  6553.6678  6576.6570  6599.6462  6622.6354 |

**Table S6.** Identified complexes in ESI Mass Spectra of reaction 40 µM MT2 with garlic compounds in ratio 1:80 after 1 h reaction at 25°C and with addition of reducers GSH, DTT and TCEP in ratio 1:100.

| **Reagent** | **Identified complex** | **Found masses (Da)** | **Calculated masses (Da)** |
| --- | --- | --- | --- |
| Ajoen | Zn_0_M_0_MT_ox_  Zn_0_M_1_MT_ox_  Zn_1_M_1_MT_ox_  Zn_2_M_0_MT  Zn_2_M_0_MT*MeOH  Zn_2_M_1_MT  Zn_3_M_1_MT_ox_  Zn_2_M_2_MT  Zn_4_M_1_MT  Zn_4_M_2_MT_ox_  Zn_4_M_3_MT_ox_  Zn_4_M_4_MT_ox_ | 6021.8966  6091.8647  6158.8758  6166.8977  6201.8674  6238.8798  6298.7840  6312.8881  6363.7162  6431.6765  6504.6808  6575.6817 | 6022.0791  6092.0551  6160.0239  6165.0544  6198.0879  6237.0544  6300.9836  6309.0544  6364.9127  6436.9127  6508.9127  6580.9127 |
| Ajoen  +  GSH | Zn_0_M_0_MT_ox_  Zn_0_M_1_MT_ox_  Zn_2_M_0_MT_ox_  Zn_3_M_0_MT_ox_  Zn_4_M_0_MT_ox_  Zn_0_M_0_MT_ox_GSH_1_  Zn_0_M_1_MT_ox_GSH_1_  Zn_0_M_0_MT_ox_GSH_2_ | 6021.8786  6088.8250  6153.7590  6221.6942  6285.6429  6326.9136  6398.9264  6637.9867 | 6022.0791  6089.1805  6153.9686  6221.9290  6285.8581  6328.9624  6398.1311  6638.3715 |
| Ajoen  +  DTT | Zn_1_M_0_MT  Zn_2_M_0_MT  Zn_2_M_0_MT*MeOH^+^  Zn_3_M_0_MT  Zn_4_M_0_MT  Zn_5_M_0_MT | 6102.9044  6164.8271  6197.8012  6230.7559  6293.6947  6358.6670 | 6101.1252  6165.0544  6198.0879  6228.9836  6292.9127  6356.8419 |
| Ajoen  +  TCEP | Zn_1_M_0_MT_ox_  Zn_2_M_0_MT  Zn_2_M_0_MT* Na^+^  Zn_3_M_0_MT  Zn_2_M_0_MT* Na^+^  Zn_4_M_0_MT | 6130.8577  6166.8419  6189.8352  6229.7761  6253.7645  6296.7407 | 6134.1587  6165.0544  6188.0436  6228.9836  6251.9728  6292.9127 |
|  |  |  |  |
| Allicin | Zn_4_M_1_MT_ox_  Zn_4_M_2_MT_ox_  Zn_4_M_3_MT_ox_  Zn_4_M_4_MT_ox_  Zn_4_M_5_MT_ox_  Zn_4_M_6_MT  Zn_4_M_7_MT  Zn_4_M_8_MT | 6359.7864  6432.7753  6504.7649  6577.7704  6649.7813  6723.7967  6796.7930  6870.8044 | 6359.8815  6432.8815  6504.8815  6577.8893  6649.8893  6724.9127  6796.9127  6868.9127 |
| Allicin  +  GSH | Zn_0_M_0_MT_ox_  Zn_0_M_1_MT_ox_  Zn_2_M_0_MT_ox_  Zn_3_M_0_MT_ox_  Zn_4_M_0_MT_ox_  Zn_0_M_4_MT  Zn_4_M_1_MT  Zn_0_M_5_MT  Zn_4_M_2_MT_ox_  Zn_4_M_3_MT_ox_  Zn_4_M_0_MT_ox_GSH_1_  Zn_4_M_1_MT_ox_GSH_1_  Zn_4_M_2_MT_ox_GSH_1_  Zn_4_M_3_MT_ox_GSH_1_  Zn_4_M_1_MT_ox_GSH_2_  Zn_4_M_2_MT_ox_GSH_2_  Zn_4_M_3_MT_ox_GSH_2_ | 6020.9630  6088.9090  6154.8714  6220.8096  6287.7314  6327.8119  6358.7178  6397.8007  6430.7122  6505.7340  6591.7770  6663.7742  6736.7933  6809.7953  6971.8573  7043.8616  7115.8682 | 6021.0713  6088.0239  6154.9764  6220.8712  6287.8737  6325.1961  6358.8659  6397.1961  6430.8659  6505.8893  6591.8632  6663.9341  6736.9419  6809.9497  6972.0257  7044.0257  7116.0302 |
| Allicin  +  DTT | Zn_1_M_0_MT_ox_*MeOH  Zn_2_M_0_MT  Zn_2_M_0_MT_ox_*MeOH  Zn_3_M_0_MT  Zn_3_M_0_MT*MeOH  Zn_4_M_0_MT  Zn_5_M_0_MT  Zn_6_M_0_MT  Zn_7_M_0_MT | 6130.9550  6164.9331  6195.8867  6229.8591  6261.8340  6292.7879  6356.7176  6421.6498  6483.5391 | 6134.1588  6165.0544  6198.0879  6228.9836  6262.0171  6292.9127  6356.8419  6420.7710  6484.7002 |
| Allicin  +  TCEP | Zn_2_M_0_MT  Zn_2_M_0_MT_ox_*MeOH  Zn_3_M_0_MT  Zn_3_M_0_MT*Na^+^  Zn_4_M_0_MT  Zn_5_M_0_MT  Zn_6_M_0_MT  Zn_7_M_0_MT | 6166.9487  6195.8783  6229.8658  6253.8408  6292.7908  6355.7266  6420.6580  6484.5591 | 6165.0544  6198.0879  6228.9836  6251.9728  6292.9127  6356.8419  6420.7710  6484.7002 |

**Table S7.** A top-down analysis identified ajoene-modified MT2 PrSMs along with their respective E-values. The modified sequence regions are indicated in parentheses and highlighted in yellow. The top PrSM spectrum (No. 1) is shown in Figure 11.

| No | PrSM sequence | E-value |
| --- | --- | --- |
| 1 | MDPNCSCAAGDSCTCAGSCKCKECKCTS(CKKSCCSCCPVGCAKCAQGCICKGA)SDKCSCCA | 1.12 · 10^-7^ |
| 2 | MDPNCSCAAGD(SCTCA)GSCKCKECKCTSCKKSCCSCCPVGCAKCAQGCICKGASDKCSCCA | 7.00 · 10^-6^ |
| 3 | MDPNCSCAAGDSCTCAGSCKCKECKCTSCKK(SCCSCCPVGCAKCAQGCICK)GASDKCSCCA | 9.66 · 10^-6^ |

**Table S8.** A top-down analysis identified allicin-modified MT2 PrSMs along with their respective E-values. The modified sequence regions are indicated in parentheses and highlighted in yellow. The top PrSM spectrum (No. 1) is shown in Figure 11.

| No | PrSM sequence | E-value |
| --- | --- | --- |
| 1 | MDPNCSCAAGDSCTCAGS(CKCKECKCTSCK)KSCCSCCPVGCAKCAQGCICKGASDKCSCCA | 1.12 · 10^-8^ |
| 2 | MDPNCSCAAGD(SCTCAGSCKCKECKCTSCKKSCCSCCPVGCAKCAQGCICK)GASDKCSCCA | 3.63 · 10^-7^ |
| 3 | MDPNCSCAAGD(SCTCAGSCKCKECKCTSCK)KSCCSCCPVGCAKCAQGCICKGASDKCSCCA | 3.66 · 10^-7^ |
| 4 | MDPNCSCAAGDSCTCAGSCKCKECKCTSCKKSCCSCCPVGCAKCAQGCICKGASDKCSC(CA) | 6.65 · 10^-7^ |
| 5 | MDPNCSCAAGDSCTCAGSCKCKECKCTSCKKSCCSCCPVGCAK(CAQGCICKGASDKCSCCA) | 6.97 · 10^-7^ |
| 6 | MDPNCSCAAGDSCTCAGSCKCKECKCTSCKKSCCSCCPVGCAKCAQGCICKGASDKCSC(CA) | 1.09 · 10^-6^ |
| 7 | MDPNCSCAAGDSCTCAGSCKCKECKCTSCKKSCCSCCPVGCAKCAQGCICKGASDKCSC(CA) | 1.13 · 10^-6^ |
| 8 | MDPNCSCAAG(DSCTCAGSCKCKECKCTSCKKSCCSCCPVGC)AKCAQGCICKGASDKCSCCA | 1.21 · 10^-6^ |
| 9 | MDPNCSCAAGD(SCTCAGSCKCKECKCTSCKKSCCSCCPVGCAKCAQGCICK)GASDKCSCCA | 1.22 · 10^-6^ |
| 10 | MDPNCSCAAGDSCTCAGSCKCKECKCT(SCKKSCCSCCPV)GCAKCAQGCICKGASDKCSCCA | 2.53 · 10^-6^ |
| 11 | MDPNCSCAAGD(SCTCAGSCKCKECKCTSCKKSCCSCCPVGCAKCAQGC)ICKGASDKCSCCA | 2.56 · 10^-6^ |
| 12 | MDPNC(SC)AAGDSCTCAGSCKCKECKCTSCKKSCCSCCPVGCAKCAQGCICKGASDKCSCCA | 3.08 · 10^-6^ |
| 13 | MDPNCSCAAGDSCTCAGSCKCKECKCTSCKKSC(CSCCPVGCAKCAQGCICK)GASDKCSCCA | 4.09 · 10^-6^ |
| 14 | MDPNCSCAAGD(SCTCAGSCKCKECKCTSCKKSCCS)CCPVGCAKCAQGCICKGASDKCSCCA | 5.50 · 10^-6^ |
| 15 | MDPNCSCAAGDSCTCAGSCKCKECKCTSCKK(SCCSCCPVGCAKCAQGCIC)KGASDKCSCCA | 5.73 · 10^-6^ |
| 16 | MDPNCSCAAGD(SCTCAGSCKCKECKCTSCKKSCCSCCPV)GCAKCAQGCICKGASDKCSCCA | 9.10 · 10^-6^ |

**References**

Albrecht F, Leontiev R, Jacob, C, Slusarenko AJ (2017) An optimized facile procedure to synthesize and purify allicin. Molecules 22:770
